# Morbidity and Mortality of Caustic Ingestion in Rural Children: Experience in a New Cardiothoracic Surgery Unit in Nigeria

**DOI:** 10.5402/2012/210632

**Published:** 2012-06-14

**Authors:** E. E. Ekpe, V. Ette

**Affiliations:** ^1^Cardiothoracic Surgery Unit, Department of Surgery, University of Uyo Teaching Hospital, PMB 1136 Uyo, Akwa Ibom, Nigeria; ^2^Otorhinolaryngology Unit, Department of Surgery, University of Uyo Teaching Hospital, PMB 1136 Uyo, Akwa Ibom, Nigeria

## Abstract

*Background*. Inspite of the fact that accidental caustic ingestion is an entirely easily preventable problem, it has however persisted in rural Nigerian communities because the commonly implicated agent which is caustic soda (sodium hydroxide, NaOH) is sold in open markets without restrictive legislations. This study aims to identify the perpetuating factors of paediatric caustic ingestion and recommend preventive measures. *Method*. Retrospective analysis of clinical records of our paediatric patients who presented following caustic ingestion between November 2006 and November 2010 was made for demography, socioeconomic status of parent(s), caustic substance ingested with amount (where known), circumstance of ingestion, means of oesophageal evaluation, treatment and outcome. *Results*. There were 16 paediatric cases of caustic ingestion during the study period with age ranging from 1 to 18 years with mode in the 1–3 years group and male : female ratio 4.3 : 1. In 100% of the cases, the caustic ingestion was accidental, while caustic soda was the agent in 93.7%, and 87.5% of the parents were into local soap and detergent production. In all patients, the oesophagus was evaluated with late barium swallow/meal and oesophagoscopy before treatment. *Conclusion*. Caustic ingestion among rural children in Nigeria can be prevented.

## 1. Introduction

The ingestion of caustic substances induces a wide range of injuries to the gastrointestinal tract, which can be mild or fatal, or leads to chronic disease and is a worldwide problem [1, 2]. Caustic ingestion in children is usually accidental ingestion, while ingestion in adults is often due to suicidal intent, and injuries tend to be more severe [1]. Approximately 17,000 ingestions involving caustic agents were reported to US poison centres in 1988, which when extrapolated to the US population yields an estimate of approximately 26,000 ingestions of corrosive agents yearly [3].

Caustic agents with a pH level <2 or >12 rapidly penetrate layers of the esophagus resulting in necrosis-induced eschar formation in the mucosa that limits deep tissue penetration. The extent of tissue destruction depends on the physical form, type, and concentration of corrosive agent, premorbid state of the tissue, contact duration, and amount of substance ingested. Esophageal mucosa is thought to be more resistant to acidic than alkaline substances, as alkaline liquids are often highly viscous and thus persist for a longer duration in the esophageal mucosa causing liquefactive necrosis, and serious esophageal injury becomes inevitable once alkaline liquids penetrate deep muscle layers [1].

Caustic ingestion by children has persisted in most developing countries including Nigeria [4–6]. Inspite of the fact that accidental caustic ingestion is an entirely easily preventable problem, [2] it has however persisted in rural Nigerian communities because the commonly implicated agent which is caustic soda (sodium hydroxide, NaOH) is sold in open markets without restrictive legislations [5]. This substance when in liquid form shares most of the characteristics of drinking water that is, colourless, odourless, and tasteless [1]. Caustic soda is used in many Nigerian homes for local soap and detergent production, a practice that is increasingly widespread. This is because the skill of soap and detergent production is regularly taught to the unemployed rural village dwellers as part of the poverty alleviation programmes of the Nigerian government. And in most rural homes engaging in this trade, facilities are insufficient to enable delineation of soap production from food processing and cooking section of the household. When solution of caustic soda is stored in such areas that are accessible to children and in familiar containers, the stage is set for accidental ingestion of such dangerous chemical.

Alarmed by the frequent presentation of rural children to our new cardiothoracic surgery unit with complaints of caustic ingestion, we set out to analyse our experience in the first four years with a view to discover the risk factors to exposure to caustic agents in the rural Nigerian communities and clinicopathologic characteristics of caustic ingestion in our paediatric patients and recommend preventive measures against accidental caustic ingestion.

## 2. Objective

It was analyse the paediatric cases of caustic ingestion in our new cardiothoracic surgery unit with a view to identify the perpetuating factors and recommend preventive measures.

## 3. Patients and Methods

This is a retrospective study of paediatric patients who presented to our unit following caustic ingestion between November 2006 and November 2010 (four years). Sources of data included admission records, patients' records, cardiothoracic surgery unit records, and theatre registers. Data extracted for analysis included patients' demographic data, parental socioeconomic data, diagnosis, caustic agent ingested including quantity (where possible), and reason for ingestion, complication, and treatment with outcome. All patients were evaluated in regard to the physical examination, routine laboratory investigations, and radiological assessment performed.

 Our unit though new and first in the state with a population of 3,902,051 people (2006 national census) is still creating awareness amongst the doctors on its existence and the range of pathologies that should be referred to it.

## 4. Results

The total number of children who presented following caustic ingestion during the study period was 16 with age ranging from one year and two months to 18 years (mean age = 5 years and 4 months). The modal age group was 1–3 years where 10 patients belonged. There were 13 boys and three girls (M : F = 4.3 : 1). The reason for caustic ingestion was accidental in all the 16 patients (100%), while 15 (93.7%) out of the 16 patients ingested caustic soda. The only ingestion of acid was in the 18-year-old daughter of a civil servant who mistakenly ingested concentrated acid stored in wine bottle in her mother's wardrobe. The highest educational attainment of the mothers of the patients was secondary education in 9 (56%) and primary education in the remaining 44%. Also 87.5% (14) of the mothers of the patients were engaged in local soap making, while one each was a civil servant and farmer, respectively. All the patients (100%) were rural dwellers.

**Figure 1:**
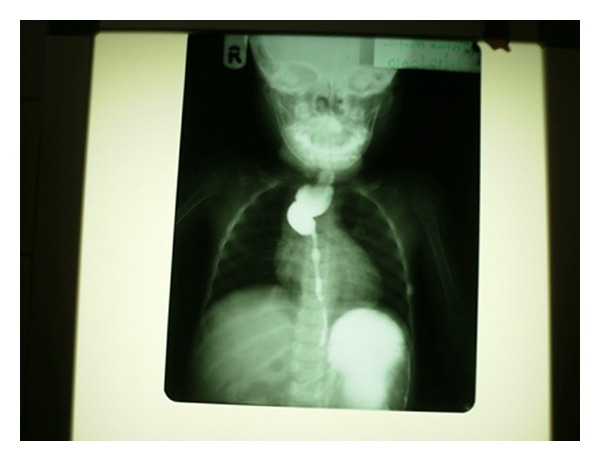
Preoperative barium swallow/meal of a child with corrosive oesophageal stricture.

**Figure 2:**
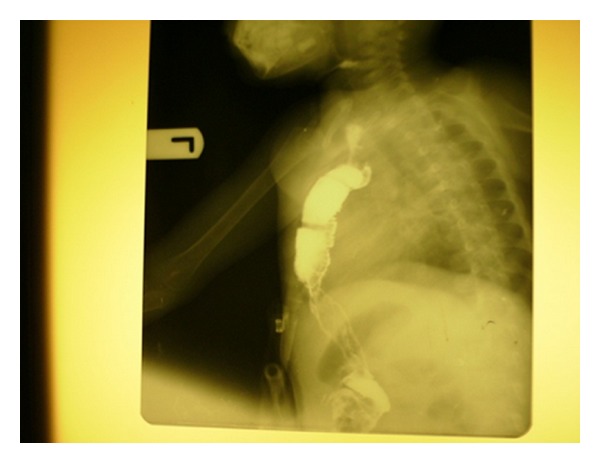
Postoperative barium swallow/meal outlining the neooesophagus in the anterior mediastinum.

Twelve (75%) out of the 16 patients presented to our unit on referral after having presented to and received initial treatment from other hospitals, while only four (25%) presented to our unit early following caustic ingestion. All patients (100%) had barium oesophagogram and late oesophagoscopy during the assessment of the oesophagus. Long-segment oesophageal stricture was present in three (18.7%) of the patients, one of which has had successful oesophageal replacement operation using colonic conduit (Figures 1 and 2), while the parents of the remaining two patients requested for referral to other centres. Barium swallow oesophagogram demonstrated normal oesophageal capacity in eight (50%) patients, and dilatable/short-segment oesophageal stricture in four (25%) patients all of whom achieved satisfactory swallowing after 2–4 sessions of oesophageal dilatation (Table 1). The only patient who ingested acid developed extensive oesophagogastroduodenal strictures and suffered from chronic protein energy malnutrition because of inadequate jejunostomy tube feeding and absence of parenteral nutrition. There was no treatment-related death.

Stamm's gastrostomy was done for seven patients due to severe dysphagia, while feeding jejunostomy was done for one patient because of extensive oesophago-gastro-duodenal strictures. Eight patients were able to swallow well after three to four days of acute-phase treatment.

## 5. Discussion

Caustic ingestion in children though still encountered globally is certainly most frequently encountered by thoracic surgeons working in the developing countries where poverty level is high, and there are no restrictions to sale and handling of caustic chemicals [2, 4–7]. The present study reveals the great impact of caustic ingestion on the overall toll of paediatric thoracic morbidity and mortality in Nigeria. This study reveals an average incidence of four cases per year in our centre. This incidence has shown that caustic ingestion in children in Nigeria may actually be increasing or the incidence in the south-south region of Nigeria where our unit is situated may be higher than elsewhere considering the incidences in the previous Nigerian studies by Ogunleye et al. which recorded 23 cases in ten years, Onotai and Nwogbo. which recorded 30 cases in ten years, and Adegboye et al. [4–6]. This increase although marginal does not portend improvement in the socioeconomic status of the common citizens who are rural dwellers. The predominance of male sex in this study (M : F = 4.3 : 1) which has been found in many other similar studies [2–8] may mean that boys explore their environment more than girls. This study also reveals that all incidences of caustic ingestion in children were accidental and in rural dwellers. Other studies have also documented accidental ingestion as the major reason for caustic ingestion in children [6, 7]. This actually means that dangerous chemicals are not stored out of reach of children probably as a result of ignorance on the part of the adults, carelessness, or lack of space and that caustic ingestion in rural children is a completely preventable problem [7]. This study shows that all but one patient ingested caustic soda (alkali) unlike in India where sulphuric acid is commonly ingested [9]. In the case of caustic soda which is used for local soapmaking by the low socio-economic households in rural Nigerian communities, space facility is commonly inadequate for the soapmaking area to be separated from food-processing section of the household. This has called for serious concern on the safety of this occupation and need for restructuring which may include creation of local community centres for local soap manufacturers to keep their raw materials and carry out soap production in such centres which should be away from homes and without children's presence. Another suggested preventive measure is the incorporation of colour and perfume in caustic soda granules by the manufacturers so that its solution will not be colourless and odourless, the two characteristics of safe drinking water which make solution of caustic soda impossible to be differentiated from water even by adults. The only acid ingestion which occurred in the grownup child was also accidental because the caustic agent was stored in a clean beverage drink bottle and kept in the cupboard, which made the dangerous substance nonsuspicious. Low level of parental educational attainment has been discovered to be directly related to the caustic ingestion by children as all the parents of the patients in this study had either primary (44%) or secondary education (56%). Even with this, we are confident that properly taught preventive measures can be understood by all normal adults. Family low socio-economic status has also been discovered to be a risk factor for caustic ingestion in children [2, 7].

This study has collaborated the fact that alkali damages oesophagus more than stomach, [1, 7] as all the patients who developed stricture following ingestion of caustic soda developed only oesophageal stricture ascertained during the late barium swallow and meal study and oesophagoscopy which were uniformly carried out on all our patients. These investigative modalities revealed long-segment oesophageal strictures in three (18.7%) patients, one of which has already had successful oesophageal replacement operation with colonic conduit (Figures 1 and 2). The other two patients with long-segment oesophageal strictures were referred to other cardiothoracic surgery centres within Nigeria on parental request. The other 25% of patients who developed short-segment dilatable strictures were successfully treated with oesophageal dilatation, giving a stricture rate of 50%. Other studies have noted oesophageal stricture rate of 19–52% in children caustic ingestion [2, 5, 8]. The only patient with extensive oesphago-gastro-duodenal strictures ingested acid and could not be salvaged because in the absence of parenteral nutrition, the initial jejunostomy tube feeding carried out could not improve her weight to make her fit for esophagogastrectomy and oesophagojejunostomy. This patient died of protein-energy malnutrition at home 12 months after caustic ingestion. The high stricture rate in this study is partly attributed to the late presentation by 75% of our patients. A common malinformed practice amongst the late presenters was the practice of induction or stimulation of vomiting after caustic ingestion. This has the potentiality of doubling the contact time which directly correlates with severity of oesophagitis and stricture. This subset of patients who were initially treated in private hospitals for various durations arrived at our unit beyond four weeks when oesophageal stricture had already developed. Early presentation would have afforded us the opportunity to treat respective patients with steroid and antibiotic to limit the severity of inflammation and infection which would reduce the severity of necrosis and subsequent stricture, [8, 10] although the benefit of steroid therapy is not supported by all [3]. Various studies have tried to evaluate the merits and demerits of the available conduits for oesophageal replacement in paediatric corrosive oesophageal stricture; [4, 7, 9, 11] however, the conduit should be able to grow and lengthen as the child grows. Colonic conduit used in this study has that capability in addition to leaving the stomach intact to perform its important function of storage. Finally, the recommended preventive measures must be simple, applicable, and straightforward for easy adoption by the rural dwellers.

## 6. Preventive Measures

- Public enlightenment of present and prospective rural dwellers who are engaged in local soap making on the dangers of caustic ingestion,
- storage of caustic agent in safe places that cannot be reached by children,
- separation of soap making from food processing and cooking area of the household,
- creation of local/community centres for local soap making,
- incorporation of colour and perfume to caustic soda granules at the point of production to make its solution to possess colour and odour and therefore distinguishable from drinking water,
- restriction on sale and handling of caustic substances.

## 7. Conclusion

Caustic ingestion among rural children in Nigeria appears to be increasing in the present research. The stricture rate of 50% contributes to the workload of cardiothoracic surgeons practicing in Nigeria. It is hoped that with widespread adoption of the preventive measures advocated by the present study, the menace can be drastically reduced if not eradicated among rural children.

## Figures and Tables

**Table 1:** Presentation, means of assessment, morbidity, treatment, and outcome of caustic ingestion in Nigerian children.

Variable	Frequency	Percent
Presentation		
Early	4	25
Late	12	75
Means of assessment		
Barium swallow/meal	16	100
Oesophagoscopy	16	100
Morbidity		
Normal oesophageal capacity	8	50
Short-segment/dilatable stricture	4	25
Long-segment/nondilatable stricture	3	18.7
Extensive oesophagogastroduodenal strictures	1	6.2
Treatment		
Supportive	8	50
Oesophageal dilatation	4	25
Referral	2	12.5
Oesophageal replacement	1	6.2
Feeding jejunostomy	1	6.2
Outcome		
Satisfactory	13	81.3
Unknown	2	12.5
Death	1	6.2
